# Naïve validity

**DOI:** 10.1007/s11229-017-1541-6

**Published:** 2017-09-27

**Authors:** Julien Murzi, Lorenzo Rossi

**Affiliations:** 1 Philosophy Department (KGW), University of Salzburg, Salzburg, Austria; 2 Munich Centre for Mathematical Philosophy, Ludwig-Maximilians University, Munich, Germany

**Keywords:** Curry’s paradox, Naïve validity, Substructural logics, Grounded validity

## Abstract

Beall and Murzi (J Philos 110(3):143–165, 2013) introduce an object-linguistic predicate for *naïve validity*, governed by intuitive principles that are inconsistent with the classical structural rules (over sufficiently expressive base theories). As a consequence, they suggest that revisionary approaches to semantic paradox must be substructural. In response to Beall and Murzi, Field (Notre Dame J Form Log 58(1):1–19, 2017) has argued that naïve validity principles do not admit of a coherent reading and that, for this reason, a non-classical solution to the semantic paradoxes need not be substructural. The aim of this paper is to respond to Field’s objections and to point to a coherent notion of validity which underwrites a coherent reading of Beall and Murzi’s principles: *grounded validity*. The notion, first introduced by Nicolai and Rossi (J Philos Log. doi:10.1007/s10992-017-9438-x, 2017), is a generalisation of Kripke’s notion of grounded truth (J Philos 72:690–716, 1975), and yields an *irreflexive* logic. While we do not advocate the adoption of a substructural logic (nor, more generally, of a revisionary approach to semantic paradox), we take the notion of naïve validity to be a legitimate semantic notion that points to genuine expressive limitations of fully structural revisionary approaches.

Consider the following naïve principles, governing a yet unspecified notion of validity:

- *Validity Proof* ($\mathsf {VP}$)   If $\psi$ follows from $\varphi$, then the argument $\langle \varphi \therefore \psi \rangle$ is valid.
- *Validity Detachment* ($\mathsf {VD}$)   $\psi$ follows from $\varphi$ and from the validity of the argument $\langle \varphi \therefore \psi \rangle$.

Let $\pi$ be a sentence equivalent to , where  is a name-forming device, $\bot$ is a constant for absurdity, and the predicate $\mathsf {Val}$ expresses the notion of validity characterised by $\mathsf {VP}$ and $\mathsf {VD}$.^1^ We may then reason thus. One first notices that $\bot$ follows from $\pi$ and , courtesy of VD. Since $\pi$ is equivalent to , this amounts to saying that $\bot$ follows from two occurrences of $\pi$. Structural contraction now allows one to conclude $\bot$ from a single occurrence of $\pi$, whence by VP  follows from the empty set of premises. By definition of $\pi$, this is a proof of $\pi$. But since $\bot$ has been shown to follow from $\pi$, Cut yields a proof of $\bot$. This is the validity Curry paradox, or v-Curry for short.

We should stress at the outset that the notion of validity that gives rise to paradox is *not* logical validity. Purely logical validity does not unrestrictedly satisfy $\mathsf {VP}$ (if Val is to express logical validity, the rule must be restricted to purely logical subproofs) and is certainly a consistent notion.^2^

While we do not advocate a non-classical approach to semantic notions,^3^ in order to investigate the v-Curry paradox and its philosophical implications, we’ll assume for the sake of argument that semantic paradoxes are to be solved via a revision of classical logic. Beall and Murzi (2013) point out that, on this assumption, if $\mathsf {Val}$ satisfies both $\mathsf {VP}$ and $\mathsf {VD}$ (or closely related principles), one of the classical *structural rules* must go. More generally, Beall and Murzi argue that the v-Curry paradox is a genuine semantic paradox and that, for this reason, if semantic paradoxes are to be solved via logical revision, such a revision should be substructural.^4^ Hartry Field (2017) has objected that ‘taken together, there is no reading of [$\mathsf {VP}$ and $\mathsf {VD}$] that should have much appeal to anyone who has absorbed the morals of both the ordinary Curry paradox and the Second Incompleteness Theorem’ (Field 2017, p. 1). For this reason, he concludes that the v-Curry paradox doesn’t call for a substructural revision of logic. Elia Zardini (2013, pp. 634–637) argues along similar lines that $\mathsf {VD}$ is incompatible with Löb’s Theorem and Gödel’s Second Incompleteness Theorem.

Our response to Field and Zardini is twofold. We first review their specific objections, and argue that they fall short of offering conclusive reasons to question the coherence of Beall and Murzi’s naïve principles for validity. In our next step, we introduce a semantic construction for naïve validity, recently developed in Nicolai and Rossi (2017), which generalises Kripke (1975)’s fixed-point construction for truth. Just like Kripke’s construction yields a theory of *grounded truth*, the construction for validity yields a theory of *grounded consequence* or *validity*—one that validates versions of Beall and Murzi’s principles. In keeping with our rejection of non-classical approaches to semantic notions, we do not endorse the notion of grounded validity. However, we argue that this notion provides a coherent reading of the naïve validity principles, that can be used to respond to Field’s and Zardini’s criticisms.

The discussion is organised as follows. Section 1 introduces the v-Curry paradox and suggests that it is a generalisation of the Knower paradox. Section 2 critically reviews Field’s and Zardini’s specific objections to the coherence of naïve validity. Section 3 introduces the notion of grounded validity and argues that it provides a coherent reading of (versions of) Beall and Murzi’s principles. Section 4 concludes.

## Introduction

This section briefly sets the scene. After some technical preliminaries (Sect. 1.1), we introduce the Knower and Curry’s paradoxes (Sect. 1.2). We then present the v-Curry paradox, and briefly introduce Beall and Murzi’s argument for $\mathsf {VP}$ and $\mathsf {VD}$ (Sect. 1.3) and Field’s preliminary discussion thereof (Sect. 1.4).

### Technical premilinaries

We consider a first-order language with identity, call it $\mathcal {L}_{V}$, whose logical vocabulary includes $\lnot$, $\wedge$, $\vee$, $\supset$, $\forall$, and $\exists$. We will only need the propositional fragments of the theories that we will consider, so we will ignore quantifiers from now on. In addition, $\mathcal {L}_{V}$ contains a propositional absurdity constant $\bot$, a propositional truth constant $\top$, and a binary predicate $\mathsf {Val}(x,y)$. Terms and formulae of $\mathcal {L}_{V}$ are defined as usual. Closed formulae are called ‘sentences’. We use lowercase latin letters (such as *s* and *t*) to range over closed terms of $\mathcal {L}_{V}$, lowercase greek letters (such as $\varphi$ and $\psi$) as schematic variables for $\mathcal {L}_{V}$-sentences, and uppercase greek letters (such as $\Gamma$ and $\Delta$) to range over finite multisets of $\mathcal {L}_{V}$-sentences.^5^ We require that theories formulated in $\mathcal {L}_{V}$ satisfy the following requirements:

- There is a function  such that for every sentence $\varphi$,  is a closed term. Informally,  can be understood as a quote-name forming device, so that  is a name of $\varphi$.
- For every open formula $\varphi (x)$ there is a term $t_{\varphi }$ such that the term  is $t_{\varphi }$, where ‘$\varphi (t_{\varphi }/x)$’ is the result of replacing every occurrence of *x* with $t_{\varphi }$ in $\varphi$.

Let $\mathcal {L}$ denote the $\mathsf {Val}$-free fragment of $\mathcal {L}_{V}$. We now recall the rules of *intuitionistic propositional logic*. We do not use the turnstile symbol $\vdash$ to denote logical consequence, but rather as a sequent arrow to axiomatise theories that will include logical as well as naïve validity-theoretical rules (plus implicit syntactic principles). For simplicity, we have opted for a single-conclusion natural deduction calculus in sequent-style, in which structural rules are explicitly formulated:^6^
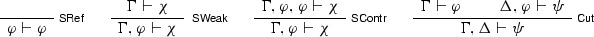

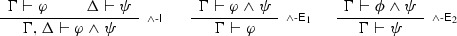

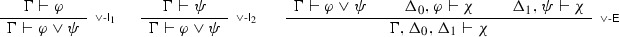

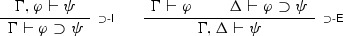

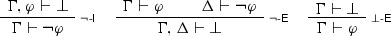
As usual, we distinguish between *structural* rules, in which no logical operator figures, and *operational* rules, which involve the occurrence of one or more logical operators.

$\mathsf {Val}(x,y)$ is to be informally understood as ‘the argument from *x* to *y* is naïvely valid’. In light of such an informal understanding, $\mathsf {Val}$ intuitively satisfies the following necessitation and factivity principles:^7^
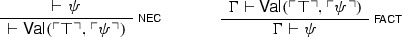
We are now in a position to present some well-known paradoxical arguments.

### The Knower and Curry’s paradox

We begin with a version of the Knower paradox (originally due to Kaplan and Montague (1960) and Myhill (1960)) formulated with our binary predicate for naïve validity. Let $\sigma$ be a sentence equivalent to . We may then reason thus. We first prove :^8^
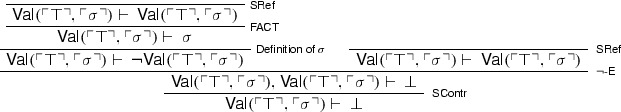


Call the above derivation $\mathcal {D}_{0}$. We then derive  from $\mathcal {D}_{0}$: 
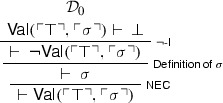


Call this derivation $\mathcal {D}_{1}$. $\mathcal {D}_{0}$ and $\mathcal {D}_{1}$ can now be combined together to yield a proof of absurdity, courtesy of $\mathsf {Cut}$: 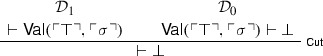
Given $\bot$-$\mathsf {E}$, the foregoing reasoning yields a proof of any sentence $\varphi$, thus making any theory in which it can be reproduced trivial.

Triviality can also be directly established without making use of $\bot$-$\mathsf {E}$, via Curry’s paradox (Curry 1942), which again we formulate by means of the naïve validity predicate. Where $\kappa$ is a sentence equivalent to , where $\psi$ is an arbitrary $\mathcal {L}_V$-sentence, one proves  reasoning in much the same way as before:
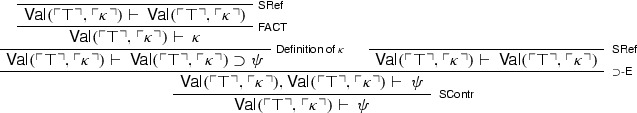


Call the above derivation $\mathcal {D}_{0}$. One then derives  from $\mathcal {D}_{0}$: 
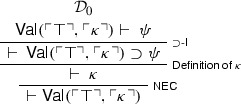
Call this derivation $\mathcal {D}_{1}$. $\mathcal {D}_{0}$ and $\mathcal {D}_{1}$ can again be combined together to yield a proof of $\psi$: 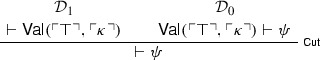


It is easy to see that the above paradoxical derivations are but variants of, respectively, the familiar Liar and Curry’s paradox, involving a *naïve truth predicate*. To see this, one need only notice that FACT is a notational variant of $\mathsf {Tr}$-E 
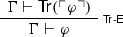
 and that NEC is but a weaker version of 
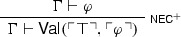
 which is in turn a notational variant of 
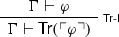
 where $\mathsf {Tr}$ expresses truth.

### The v-Curry paradox

While both the Knower and Curry’s paradoxes can be blocked by rejecting some of the standard I- and E-rules for $\lnot$ and $\supset$, there are closely related paradoxical arguments employing generalisations of $\mathsf {NEC}$ and $\mathsf {FACT}$ that cannot be so dismissed. Consider again $\mathsf {NEC}$: 
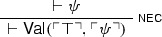
 On the naïve reading of $\mathsf {Val}$, the rule tells us that if we have proved $\psi$, i.e. if we have derived it from no assumptions, then it follows from $\top$ (which is always provable), i.e. $\psi$ follows from any sentence. A natural way to generalise $\mathsf {NEC}$, then, is to apply the validity predicate not only when a sentence has been proved, but also when a sentence has been derived from a sentence, encoding this information into the naïve validity predicate. In short, $\mathsf {NEC}$ can be liberalised to arbitrary inferences: 
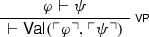
 After all, one might reason, if one wishes to express (in the object-language) that a sentence follows from the empty set of premisses, why shouldn’t one want to express in the same fashion that a sentence follows from another sentence? Indeed, an even more liberal way of expressing inferences via the naïve validity predicate would allow arbitrary side sentences, as in the following rule: 
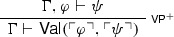


$\mathsf {FACT}$ also admits of a generalisation along similar lines: 
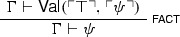
If one can derive that $\psi$ follows from $\top$ given $\Gamma$, then one can conclude that $\psi$ also follows from $\Gamma$. A straightforward generalisation can be motivated by asking what can be concluded when $\psi$ follows from an arbitrary sentence $\varphi$ (given $\Gamma$), rather than from $\top$. Suppose that $\psi$ follows from $\varphi$ given $\Gamma$: since $\psi$ is the conclusion of a chain of inferences, it is natural to ask under which conditions one can conclude $\psi$. The following (naïve) option presents itself: since $\psi$ follows from $\varphi$, if one has strong enough grounds to conclude $\varphi$, then one can combine those grounds with $\Gamma$ and derive $\psi$. In other words, the following rule is a generalisation of $\mathsf {FACT}$: 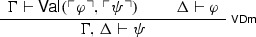 As above, there seem to be no reasons to think that the case  is conceptually different from the case .^9^

It is important to notice that $\mathsf {VDm}$ and 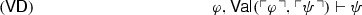
are not quite the same rule: in the terminology of Ripley (2012), $\mathsf {VD}$ is an *inference*, namely an object of the form $\Gamma \vdash \varphi$, and $\mathsf {VDm}$ is a *meta-inference*, namely a rule that allows one to derive an inference from one or more inferences. $\mathsf {VD}$ can be immediately obtained from $\mathsf {VDm}$ in the presence of the structural rule of *reflexivity*: 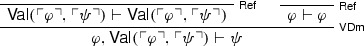 Likewise, $\mathsf {VDm}$ can be derived from $\mathsf {VD}$ given Cut. The structural difference between $\mathsf {VDm}$ and $\mathsf {VD}$ matters in a substructural setting. For instance, approaches restricting $\mathsf {Cut}$ cannot accept $\mathsf {VDm}$, since together with $\mathsf {VP}$ it makes $\mathsf {Cut}$ admissible. In Sect. 3, we will present a reading for the validity predicate that makes gives a coherent reading of $\mathsf {VDm}$ but not of $\mathsf {VD}$.

With $\mathsf {VP}$ and $\mathsf {VDm}$ (or $\mathsf {VD}$) in place, we can now introduce Beall and Murzi’s v-Curry paradox. Where $\pi$ is a sentence equivalent to  (so that $\pi$ says of itself that it entails absurdity), let $\mathcal {D}$ be the following derivation of : 
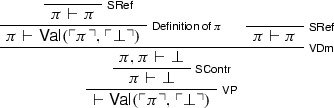
 Using $\mathcal {D}$, we can then ‘prove’ $\bot$: 
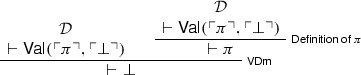
 This is the v-Curry paradox (Beall and Murzi 2013). Given that $\mathsf {VD}$ is derivable from $\mathsf {Ref}$ and $\mathsf {VDm}$, a proof of the paradox could also be given using $\mathsf {VD}$ and $\mathsf {Cut}$.

Since the argument makes no assumptions about the logic of negation and the conditional, it resists *fully structural* revisionary treatments, i.e. treatments that retain all of SRef, SContr, and Cut. In particular, paracomplete theories, which restrict the Law of Excluded Middle

$$\begin{aligned}&(\mathsf {LEM})& \psi \vdash \varphi \vee \lnot \varphi \end{aligned}$$

as well as $\lnot$-I and $\supset$-I,^10^ and standard paraconsistent theories, which restrict the principle of explosion (or *ex contraditione quodlibet*)

$$\begin{aligned}&(\mathsf {ECQ})& \varphi \wedge \lnot \varphi \vdash \psi , \end{aligned}$$

as well as $\lnot$-E and $\supset$-E,^11^ cannot be nontrivially closed under $\mathsf {VP}$ and $\mathsf {VDm}$. These theories can validate naïve semantic principles such as NEC and FACT, but they cannot be closed under their generalisations VP and $\mathsf {VDm}$, on pain of triviality. Beall and Murzi (2013) conclude from this observation that, if the semantic paradoxes are to be solved via logical revision, then one of SRef, SContr, and Cut, must go. Field disagrees.

### Field on the V-Schema

In a nutshell, Field (2017) argues that there is no coherent reading of $\vdash$ and Val for which both $\mathsf {VP}$ and $\mathsf {VDm}$ (or $\mathsf {VD}$) hold.^12^ According to Field, validity is standardly defined in one of three ways: as necessary truth-preservation, as preservation of truth-in-a-model (for suitably chosen models), or as derivability-in-*S* (for a suitably chosen formal system *S*). However, Field argues that none of these notions makes both of $\mathsf {VP}$ and $\mathsf {VDm}$ coherent. We discuss Field’s argument in detail in Sect. 2 below. We first focus on what he has to say about the V-Schema, a naïve validity principle that Beall and Murzi take to justify $\mathsf {VP}$ and $\mathsf {VD}$.

Field strongly argues against the coherence of the V-Schema
a principle that Beall and Murzi take to be as intuitive for Val as the T-Schema 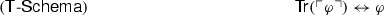
is for truth. Field rectifies the claim, advanced in Beall and Murzi (2013), that the $\mathsf {V}$-$\mathsf {Schema}$ is equivalent to (i.e. interderivable with) $\mathsf {VP}$ and $\mathsf {VD}$, since the $\mathsf {V}$-$\mathsf {Schema}$ is weaker than $\mathsf {VP}$ and $\mathsf {VD}$ taken together. He interprets Beall and Murzi as suggesting that $\mathsf {Val}$ is better understood as ‘simply a rendering of ‘$\vdash$’ into the object language (thereby allowing it to freely embed)’ (Field 2017, p. 7). But while he concedes that ‘prima facie this is a very natural suggestion’ he argues that it doesn’t support a coherent reading of the V-Schema:

Beall and Murzi’s likening of the $(\mathsf {V}$-$\mathsf {Schema}$) to the truth schema [$\ldots$] seems incorrect: even on the assumption that ‘$\vdash$’ represents a kind of validity and ‘$\mathsf {Val}$’ the same kind of validity, their schema has a ‘double occurrence of validity’ (‘$\vdash \mathsf {Val}$’) on the left side and a ‘single occurrence’ (‘$\vdash$’) on the right, making the argument from right to left [$\ldots$] problematic. And without the assumption that ‘$\vdash$’ represents a kind of validity and ‘$\mathsf {Val}$’ the same kind of validity, there seems even less reason to accept $\mathsf {VP}$. (Field 2017, p. 7)

Field then mentions a possible strengthening of $\mathsf {V}$-$\mathsf {Schema}$—one that, *given* SRef, actually delivers both $\mathsf {VP}$ and $\mathsf {VD}$: 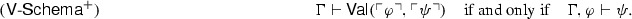
However, Field also dismisses the $\mathsf {V}$-$\mathsf {Schema^{+}}$, on the grounds of cases such as the following:

1 $$\begin{aligned}&\text{ snow } \text{ is } \text{ white, } \text{ grass } \text{ is } \text{ green } \vdash \text{ snow } \text{ is } \text{ white, } \end{aligned}$$

2 $$\begin{aligned}&\text{ snow } \text{ is } \text{ white } \vdash \mathsf {Val}(`\text {grass is green'},`\text {snow is white'}). \end{aligned}$$

According to Field, (1) holds, but (2) doesn’t.

Field’s argument fails to convince, however. To be sure, both the $\mathsf {V}$-$\mathsf {Schema}$ and the $\mathsf {V}$-$\mathsf {Schema}^{+}$ fail if $\mathsf {Val}$ is interpreted as expressing *logical* validity. However, such a reading is already known to be unsuitable for $\mathsf {VP}$ (Ketland 2012; Cook 2014; Nicolai and Rossi 2017, §2). Hence, *a fortiori*, it does not fit stronger principles such as the $\mathsf{V}\hbox {-}\mathsf{Schema}$ and the $\mathsf {V}$-$\mathsf {Schema}^{+}$. In any event, absent a precise characterisation of $\vdash$ and $\mathsf {Val}$, it is unclear whether one should accept or reject (1) and (2), and the $\mathsf{V}\hbox {-}\mathsf{Schema}$ and the $\mathsf {V}$-$\mathsf {Schema}^{+}$ more generally. Field contends that no coherent notion of validity simultaneously satisfies Beall and Murzi’s principles. We aim to show otherwise.

## The case against VP and VD

We now turn to Field’s positive case for claiming that there is a fundamental asymmetry between truth-theoretical and naïve validity-theoretical principles. We first discuss two classicality constraints for $\mathsf {Val}$, which Field expresses sympathy for but doesn’t endorse (Sect. 2.1). We then turn to Field’s argument from definability, that the standard ways of defining validity are incompatible with at least one between VP and VD (Sect. 2.2).

### Classicality constraints

Field (2017, pp. 8–9) considers two possible classicality constraints for $\mathsf {Val}$:

*Weak Classicality Constraint (WCC)* If the $\mathsf {Val}$-free fragment of $\mathcal {L}_{V}$ is classical, then sentences containing $\mathsf {Val}$ (restricted to inferences in $\mathcal {L}$) should also be classical, in the sense of obeying classical laws like excluded middle and explosion.

*Strong Classicality Constraint (SCC)* *Even for non-classical* [$\mathsf {Val}$-free] *languages* $\mathcal {L}$, $\mathsf {Val}$ (applied to $\mathcal {L}$) should be a classical predicate, in the sense that classical laws like excluded middle and explosion apply to sentences containing it.

In Field’s view, both principles are incompatible with a naïve conception of validity. As he writes, the weaker principle ‘would immediately rule out substructural solutions to the validity paradoxes in otherwise classical languages’ Field (2017, p. 8). What is more, Field maintains that WCC also rules out non-classical solutions to Knower-like paradoxes generated using $\mathsf {NEC}$ and $\mathsf {FACT}$. But why should validity be classically constrained? Field mentions two possible arguments.

First, given that ‘the notion of validity should serve as a regulator of reasoning’, Field argues that it ‘would seem as it would hamper that role if there were inferences for which we had to reject that they were either valid or not valid (or accept that they were both) $[\ldots ]$’ (Field 2017, p. 9). Second, Field mentions what he calls the *hypocrisy problem*. He argues that if validity were non-classical, one would have to formulate a theory of validity within a non-classical meta-theory. But because it is very hard to give a non-classical meta-theory, one might as well endorse one of WCC and SCC, thus avoiding the hypocrisy problem.

Some comments are in order. First, on the rejection of classical laws for naïve validity, it is unclear why this should be more problematic than a departure from classical logic in the case of *truth*. After all, truth would also appear to regulate reasoning—for instance, it is widely held that assertion aims at truth (see e.g. Dummett 1959). Second, WCC and SCC are strictly speaking not incompatible with a naïve view of validity. The reason is that, while WCC and SCC would force $\mathsf {Val}$ to satisfy both the excluded middleand explosionsome substructural approaches, such as (validity-theoretic versions of) the theories in Zardini (2011) and Ripley (2012), validate versions of both principles, for the whole language.

To be sure, WCC and SCC might be construed as requiring that sentences containing $\mathsf {Val}$ behave fully classically, where this includes the satisfaction of the structural rules. This is where WCC and SCC part ways, however. If one interprets WCC in this more stringent way, the criterion is still satisfied by several substructural theories of naïve validity, including the approach of Ripley (2012) and the theory developed in Nicolai and Rossi (2017), which will be also described in Sect. 3. Just as in the case of many non-classical theories of truth, in such theories the $\mathsf {Val}$-free sentences (and also some sentences featuring $\mathsf {Val}$) satisfy all classical rules, operational and structural alike. By contrast, SCC is incompatible with substructural approaches that validate $\mathsf {VP}$ and $\mathsf {VDm}$. However, in absence of a plausible independent reason to accept SCC (in its stricter reading), this requirement simply begs the question against substructural logicians who are such because of the v-Curry and related paradoxes.

### Field’s argument from definability

Field merely expresses sympathy towards WCC and SCC: his main argument against the coherence of naïve validity-theoretical principles is independent of either principle. In a nutshell, the argument is that none of the three main accounts of validity (validity as necessary truth-preservation, validity as preservation of truth-in-$\mathfrak {M}$, and validity as provability-in-*S*) is naïve. Hence, pending an alternative reading of Val, there seems to be no good reason to accept both of $\mathsf {VP}$ and $\mathsf {VDm}$.

#### Validity as necessary truth-preservation

Suppose that validity is equated with necessary truth-preservation, in the following sense:

($$\mathsf {VTP}$$)
The argument $\Gamma \therefore \varphi$ is valid if and only if necessarily, if all the $\psi \in \Gamma$ are true, then $\varphi$ is also true.

On this view, Field argues, one between $\mathsf {VP}$ and $\mathsf {VD}$ must fail. For ‘any paradoxes of validity will simply be paradoxes of truth in the modal language. Standard resolutions of the paradoxes of truth $\ldots$ [will] carry over’ (Field 2017, p. 10). Thus, Field concludes, ‘Beall and Murzi’s idea that there are *new* paradoxes of validity $\ldots$ requires rejecting this reduction of validity to truth and $\ldots$ modality’ (*ibid.*).

One first difficulty with the argument is that, on a natural reading of it, it seems premised on a *standard revisionary approach*, i.e. one validating the structural rules of $\mathsf {SRef}$, $\mathsf {SContr}$, and $\mathsf {Cut}$. But such rules are *incompatible* with naïve validity. Presumably, then, Field intends the argument to establish that standard paracomplete and paraconsistent approaches can already cope with the v-Curry paradox, if $\mathsf {VTP}$ holds. But there are difficulties with this suggestion, too. As Field (2008, pp. 42–43, pp. 284–286, and pp. 377–378) has long pointed out, $\mathsf {VTP}$ cannot be consistently asserted in a fully structural setting, on pain of Curry-driven triviality.^13^ But then, $\mathsf {VTP}$ cannot be used to show that fully structural revisionary theorists have a reason to invalidate one between $\mathsf {VP}$ and $\mathsf {VD}$: such theorists *reject* $\mathsf {VTP}$.

Field’s argument may be recast as the contention that fully structural solutions that invalidate $\supset$-I can reject $\mathsf {VP}$, and that fully structural solutions that invalidate $\supset$-E can reject $\mathsf {VDm}$ and $\mathsf {VD}$. However, this observation by itself does not tell against proponents of naïve validity. Substructural theorists who are such because of the v-Curry paradox can retort that they can offer a more compelling package: they can not only retain each of $\supset$-I, $\supset$-E, $\mathsf {VP}$ and either $\mathsf {VDm}$ or $\mathsf {VD}$; they can also consistently assert (suitable versions of) $\mathsf {VTP}$ (see Murzi and Shapiro 2015).

#### Validity as preservation of truth-in-$\mathfrak {M}$

Field considers various possible model-theoretic characterisations of validity. Where $\mathcal {L}$ is a language mathematically rich enough to formulate Peano Arithmetic (PA) or Zermelo-Fraenkel set theory (ZF), he observes that validity can be defined model-theoretically. As he writes,

[f]ocusing on one-premise inferences, the general form [of these definitions] is either (i) that the inference from $\varphi$ to $\psi$ is valid if and only if in all models $\mathfrak {M}$ of type $\Psi$, if $\varphi$ has a designated value in $\mathfrak {M}$ then so does $\psi$; or (ii) that it is valid if and only if in all models $\mathfrak {M}$ of type $\Psi$, the value of $\varphi$ is less or equal to that of $\psi$. (Field 2017, p. 17; Field’s notation has been adapted to ours)

Field’s general point is that in each of these cases, validity cannot be paradoxical on the grounds that ‘the notion of validity is to be literally defined in set theory’ (Field 2008, p. 298).

The argument fails to convince, however. If it were legitimate to assume that validity is model-theoretically definable in order to show that there are no paradoxes of naïve validity, then it would also be legitimate to assume that *truth* is model-theoretically definable in order to show that there are no paradoxes of naïve truth. But this seems unacceptable (Murzi 2014, pp. 77–8). As proponents of naïve theories of truth point out, what holds for model-relative notions need not hold for the corresponding model-independent notions (see e.g. Field 2007, p. 107). To be sure, Field might object that there is no coherent model-independent notion of naïve validity. However, his argument from model-theoretic definability does not establish this stronger conclusion.

#### Validity as provability-in-*S*

Let *S* be a consistent, recursively axiomatisable theory (formulated in $\mathcal {L}_{V}$, or in a language that extends it) that is strong enough to simulate self-reference. For simplicity, we could require that *S* interprets $\mathsf {PA}$ or $\mathsf {ZF}$. Either way, the notion of derivability in *S*, in symbols $\vdash _{S}$, is also a recursively enumerable relation. Field (2017, p. 12) suggests that *S* might be taken to be a ‘mathematical theory $\ldots$ identical to that we use in our informal reasoning’ , whose consequence relation $\vdash _{S}$ plausibly models the notion of *validity* associated with *S*, or at least one such notion. If the validity predicate $\mathsf {Val}(x, y)$ is to express $\vdash _S$ in the object-language, then it is natural to interpret $\mathsf {Val}(x,y)$ as *derivability in S*. To indicate this specific reading, and in this subsection only, we will write $\mathsf {Val}_{S}(x,y)$. But here lies the problem.

If *S* is closed under $\mathsf {VDm}$ or $\mathsf {VD}$, one can now derive all instances of the following schema:3But since $\mathsf {Val}_{S}$ now expresses derivability in *S*, one can use $\mathsf {Val}_{S}$ to define a (standard) provability predicate $\mathsf {Prov}_{S}(x)$ that provably applies to the code of $\varphi$ if there is a proof of $\varphi$ in *S*. That is,  becomes equivalent to . However, (3) entails in *S* every instance of the local reflection principle , and therefore by Löb’s Theorem (or Gödel’s Second Incompleteness Theorem) that *S* is trivial (see Boolos (1993), Ch. 3). On these grounds, Field and Zardini reject $\mathsf {VDm}$ and $\mathsf {VD}$. As Field puts it:

[g]iven that $\mathsf {PA}$ and $\mathsf {ZF}$ are presumably consistent, we must reject $\mathsf {VD}$ [...]. That, I assume, is a fact that we have come to terms with long ago. (Field 2017, p. 12)

Likewise, Zardini argues that

*derivabilty* in $\mathsf {PA}$ actually coincides with *validity* relative to $\mathsf {PA}$. It then becomes utterly unclear why, in view of these facts, one should still expect $\mathsf {VD}$ to be correct for $\mathsf {Val}$. (Zardini 2013, p. 636)

If validity is derivability in a recursively enumerable system, $\mathsf {VDm}$ and $\mathsf {VD}$ must fail.

There are some difficulties with the foregoing argument, however. Even conceding Field’s and Zardini’s assumption that naïve validity can be equated with validity *relative to* *S*, it is not at all clear that the latter notion can be identified with *derivability in* *S*. A well-known argument from the First Incompleteness Theorem, first given (as far as we know) by John Myhill (1960, pp. 466–7), suggests that validity outstrips derivability in any recursively axiomatisable theory that interprets a small amount of arithmetic, and whose axioms and rules we can at least implicitly accept as correct.

To see this, notice that we can establish *S*’s (canonical) Gödel sentence $\rho$ by means of a *valid* argument which—the First Incompleteness Theorem tells us—cannot itself be formalised in *S*. Add to *S* all instances of the local reflection principle  for *S*. Call the resulting theory $S'$. It is then a routine exercise to prove $\rho$ in $S'$. But while $S'$ proves $\rho$, it is arguable that $S'$ only articulates commitments that were already implicit in one’s acceptance of *S*. After all, it would be hard to accept *S* without accepting that it is *sound*, i.e. that what it proves holds. And yet, this is precisely what one’s acceptance of  amounts to. But then, validity relative to *S* cannot in general be identified with derivability in *S*. As Myhill puts it:

[i]t is possible to prove [$\kappa$] by methods which we must admit to be correct if we admit that the methods available in [*S*] are correct. (Myhill 1960, pp. 466–7)

From this perspective, the notion of validity that arises from $\mathsf {PA}$, $\mathsf {ZF}$, or indeed any sufficiently expressive, recursively axiomatisable theory *S* is not identifiable with the corresponding notion of derivability. While the latter is classically expressible in the target theory and fails to respect $\mathsf {VDm}$ and $\mathsf {VD}$, the former requires methods and tools that extend the target theory, such as local reflection principles.

The natural upshot of the foregoing picture is a hierarchy of ever stronger theories, none of which validates $\mathsf {VDm}$ or $\mathsf {VD}$. Suppose, following Myhill, that explicating the notion of validity relative to *S* commits one to accepting $S'$. Since Myhill’s argument does not only apply to *S* but applies equally well to $S'$, one is naturally led to accept $S''$, the theory that results from the addition of all the instances of the local reflection principles for $S'$ to $S'$. By the same token, one is led to accept the similarly defined theory $S'''$, and then to accept $S''''$, and so forth. This progression can be extended into the transfinite.^14^ There are several choices to be made when generating such a transfinite sequence of theories. Such progressions vary depending on the starting theory and on how the iterations are defined. What matters for present purposes is that such progressions have two relevant possible outcomes:

(i) The progression reaches a *halting point*, namely a theory $S^{\mathsf {H}}$ such that the progression technique that was adopted at the outset cannot be applied to $S^{\mathsf {H}}$ to yield a stronger theory that is (computationally) simple enough for Löb’s Theorem to apply.^15^
(ii) The progression reaches a stage (which may or may not be its halting point) such that the theories *beyond that stage* are too complex for Löb’s Theorem to apply.

In situations of type (i), it can be argued that the fact that $S^{\mathsf {H}}$ is a halting point is merely a technical matter, that should have no conceptual consequences. That is, one might insist that, if one accepts $S^{\mathsf {H}}$, one should also accept that it is sound, or that its proof procedures are correct. It must then be possible to prove its Gödel sentence and extend the theory, even though such extension must follow a different pattern than the progression that led from *S* to $S^{\mathsf {H}}$. Eventually, though, the iteration procedures that are needed to express the soundness of higher and higher levels of iterations will deliver theories that are too complex for Löb’s theorem to apply. Therefore, situations of type (i) collapse into situations of type (ii).

However, not even highly complex iterations to which Löb’s Theorem doesn’t apply offer positive reasons for accepting $\mathsf {VDm}$ or $\mathsf {VD}$. The problem is that even in the case of theories that are too complex to have a workable provability predicate, it is unclear that anything like $\mathsf {VDm}$ or $\mathsf {VD}$ is fully justified. In the construal of validity we are considering, namely validity relative to a theory *S*, there is no point, in any progression of theories along the lines sketched above, at which a theory $S^{\star }$ is *closed under* the local reflection principle *for* $S^{\star }$. $\mathsf {VP}$ and $\mathsf {VDm}$ or $\mathsf {VD}$ are a sort of unattainable ‘limit’ of the notions of validity relative to a theory that the acceptance of Myhill’s argument suggests—a limit that fuels the progression of theories but that remains always one step beyond the reach of every theory so generated.

#### Hierarchical validity

We have argued that understanding validity relative to *S* via a progression of ever stronger theories doesn’t validate Beall and Murzi’s naïve principles. This should not be surprising: a similar situation arises in the context of progressions of truth-theoretic principles, namely *Tarskian hierarchies*.^16^ Field (2017, §9) sketches possible hierarchical versions of $\mathsf {VP}$ and $\mathsf {VD}$. Nicolai and Rossi (2017, §2.4) provide a precise regimentation of a hierarchy for validity, and study its relation with a progression of local reflection principles. As it turns out, at each ordinal stage, the theories in the hierarchy for validity are interpretable in the theories resulting from the progression of local reflection principles. But while an iterative conception of validity does not yield the non-stratified $\mathsf {VP}$ and $\mathsf {VDm}$, it nevertheless points in a more promising direction, as Field himself suggests. Here’s how he closes his paper:

The thought might be that just as Kripke (1975) showed how to transcend the Tarski hierarchy in a non-classical setting (introducing a single unstratified non-classical truth predicate [...]), we should do the same for validity in a non-classical setting. Extending the analogy, the idea might be to argue in a non-classical setting that by starting from a hierarchy of validity predicates and allowing sentences to ‘seek their own level’, an unstratified predicate that satisfied $\mathsf {VP}$ and $\mathsf {VD}$ would emerge at some fixed point. [$\dots$] Obviously there’s no way that anything like this could happen if the non-classical setting were merely paracomplete or paraconsistent, with standard structural rules—[$\ldots$] the whole point of the v-Curry argument was that mere paracompleteness or paraconsistency don’t suffice to allow for $\mathsf {VP}$ and $\mathsf {VD}$ together. But perhaps if we did a construction modeled after Kripke’s in a substructural setting, $\mathsf {VP}$ and $\mathsf {VD}$ together would emerge? That would certainly be interesting if it could be done, but Beall and Murzi don’t claim it can, and nothing in their paper gives any reason to think that it can. (Field 2017, pp. 15–6)

*But it can*. Nicolai and Rossi (2017, §§3–4) develop a construction that is in effect a naïve validity-theoretical generalisation of Kripke ’s (1975) construction for truth. Their construction, called ‘KV-construction’ (for ‘Kripke’ and ‘validity’), delivers non-trivial models of $\mathcal {L}_{V}$ (or languages extending it) where $\mathsf {VP}$ and $\mathsf {VDm}$ (together with the $\mathsf {V}$-$\mathsf {Schema}^{+}$) hold unrestrictedly. The significance of this result is not only technical: the construction can also be used to meet Field’s challenge of finding a coherent reading of the naïve validity-theoretical principles.^17^

## A Kripkean construction for naïve validity

We begin by offering a (largely informal) presentation of the KV-construction in Sect. 3.1.^18^ We then argue in Sect. 3.2 that one of the models that results from the KV-construction suggests a coherent interpretation of naïve validity: *grounded validity*.

### The KV-construction

The KV-construction generalises Kripke’s treatment of truth (strong Kleene version) to naïve validity. Rather than constructing successions of sets of sentences (leading to a fixed point), it builds successions of sets of inferences or sequents. We work with the language of arithmetic, enriched with a primitive binary predicate $\mathsf {Val}(x,y)$, for validity; we call this language $\mathcal {L}^{a}_{V}$. More precisely, the KV-construction generalises inferences to multiple-conclusion $\mathcal {L}^{a}_{V}$-sequents, i.e. objects of the form $\Gamma \vdash \Delta$, where both $\Gamma$ and $\Delta$ are finite sets of $\mathcal {L}^{a}_{V}$-sentences. From now on, we will work with *finite sets* rather than multisets. We will continue using capital Greek letters (such as $\Gamma$ and $\Delta$) to denote finite sets.

The starting point of the KV-construction is analogous to Kripke’s: we take the extension of $\mathsf {Val}$ to be momentarily empty, and ‘fill’ it gradually. When some inferences are accepted, they can be declared ‘naïvely valid’ with the introduction of $\mathsf {Val}$. In Kripke’s construction, arithmetical truths and falsities are used to start off the interpretation of the truth predicate. An analogous starting point is available for sequents. The standard model $\mathbb {N}$ also provides arithmetical inferences (i.e. not involving the validity predicate):

$$\begin{aligned} \text{ the } \text{ sequents } \Gamma \vdash \varphi , \Delta&\text{ where } \varphi \text{ is } \text{ an } \text{ atomic } \text{ arithmetical } \text{ sentence } \text{ and } \mathbb {N} \models \varphi ,\\ \text{ the } \text{ sequents } \Gamma , \psi \vdash \Delta&\text{ where } \psi \text{ is } \text{ an } \text{ atomic } \text{ arithmetical } \text{ sentence } \text{ and } \mathbb {N} \not \models \psi \end{aligned}$$

That is, we start from inferences leading to an arithmetical truth, or starting from an arithmetical falsity, with arbitrary side sentences.

We now need to explain how the acceptance of a collection of sequents can lead to the acceptance of other sequents. Since we are dealing with sequents, and not with sentences, this cannot happen (as in Kripke’s case) via some evaluation scheme. However, we can resort to *meta-inferences*, namely principles that determine which sequents are to be accepted given the acceptance of one or more other sequents. In the KV-construction, we can consistently use inductive clauses modelled after *all the classical meta-inferences*. Of course, we need to devise clauses for the validity predicate too, namely clauses that tell us when a sentence of the form  can be introduced in a sequent, given some previously accepted sequents. An inspection of the naïve principles for validity suggests an obvious option: these principles are classical *implication* principles formulated using a predicate, namely $\mathsf {Val}$, rather than a connective. It is then natural to use meta-inferences for $\mathsf {Val}$ modelled after the classical meta-inferences adopted to introduce conditionals in sequents.

Several formalisms can be used to capture meta-inferences; we select a variant of a classical *sequent calculus*. We now introduce the clauses that determine the acceptance of new sequents.^19^ As in Kripke’s construction, we express them via an operator (on sequents rather than sentences), which we call $\Psi$. $\Psi$ takes a set of sequents *S* and adds to it the sequents with arithmetical atomic truths in the consequent, or arithmetical atomic falsities in the antecedent, and the sequents resulting by applying the remaining clauses to the sequents in *S*. For *S* a set of sequents, $\Gamma \vdash \Delta$ is in $\Psi (S)$ if: 
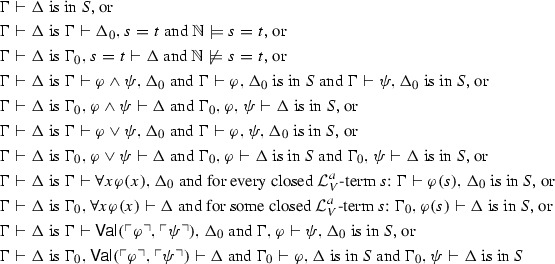
Taking $\varnothing$ for *S*, we generate a set $\Psi (\varnothing )$ that contains all the sequents with atomic arithmetical truths in their consequent, or with atomic arithmetical falsities in their antecedent, and nothing else. Further iterations of $\Psi$ lead to growing sets of inferences, that match Kripke’s sequence of pairs of sets. We index the stages of this progression with ordinals, writing $S^{\alpha }_{\Psi }$ for the $\alpha$-th iteration of $\Psi$ applied to *S*. In general, the sequence is defined as follows, for every set of sequents *S*, and $\delta$ a limit ordinal:

$$\begin{aligned}&S^{\alpha +1}_{\Psi } := \Psi (S^{\alpha }_{\Psi })&S^{\delta }_{\Psi } := \bigcup _{\alpha <\delta }S^{\alpha }_{\Psi } \end{aligned}$$

The KV-construction also has fixed points. That is, there is an ordinal $\zeta$ such that, for every set of sequents *S*:

$$\begin{aligned} S^{\zeta +1}_{\Psi } = \Psi (S_{\Psi }^{\zeta }) = S_{\Psi }^{\zeta } \end{aligned}$$

We indicate with $S_{\Psi }$ the fixed point of $\Psi$ generated by *S*, and with $\mathsf {I}_{\Psi }$ the fixed point of $\Psi$ generated by $\varnothing$. $\mathsf {I}_{\Psi }$ is the least fixed point of the KV-construction, as it is included in every other such fixed point.

$\mathsf {I}_{\Psi }$ validates versions of $\mathsf {VP}$, $\mathsf {VDm}$, the $\mathsf {V}$-$\mathsf {Schema}$, and the $\mathsf {V}$-$\mathsf {Schema}^{+}$. For $\varphi , \psi$ sentences of $\mathcal {L}^{a}_{V}$, and $\Gamma , \Delta$ finite sets of $\mathcal {L}^{a}_{V}$-sentences, the following holds:
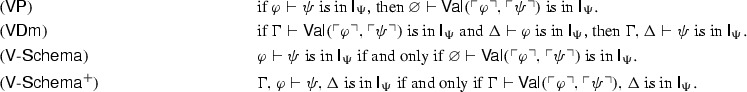
Thus, $\mathsf {I}_{\Psi }$ validates all of Beall and Murzi’s naïve principles for validity, with the only exception of $\mathsf {VD}$ (more on this in Sect. 3.2.2). In addition, all the classical structural rules bar reflexivity are recovered in $\mathsf {I}_{\Psi }$: this fixed point is closed under clauses expressing left and right contraction, left and right weakening, and cut. All the results we mentioned about $\mathsf {I}_{\Psi }$ can be extended to fixed points including $\mathsf {I}_{\Psi }$, but this would require some non-trivial extra work (see Nicolai and Rossi 2017, §4.2). A fixed point $S_{\Psi }$ can thus be used to define a model of the full language $\mathcal {L}^{a}_{V}$, where all of $\mathsf {VP}$, $\mathsf {VDm}$, the $\mathsf {V}$-$\mathsf {Schema}$, and the $\mathsf {V}$-$\mathsf {Schema}^{+}$ hold. The extension of the validity predicate determined by $S_{\Psi }$ is given by the sequents of the form $\varphi \vdash \psi$ in $S_{\Psi }$.

We conclude this section by noticing that the computational complexity of $\mathsf {I}_{\Psi }$ is identical to the computational complexity of the least Kripke fixed point for truth—a relatively low complexity in the context of semantic theories of truth. We also observe that, just as in the case of Kripke’s theory, the clauses of the definition of $\Psi$ can be turned into a recursively enumerable theory that axiomatizes adequately, in the sense of Fischer et al. (2015), the set of the fixed points extending $\mathsf {I}_{\Psi }$. Naïve validity need not be too complicated to reason with.

### Grounded validity

$\mathsf {I}_{\Psi }$ provides a coherent reading of the notion of validity—one that makes sense of many of the naïve principles discussed in Beall and Murzi (2013). Following Kripke’s construction, we call this reading *grounded validity*, i.e. validity as grounded in truths and falsities of the base language.^20^ The idea of grounded validity is simple: a sequent $\Gamma \vdash \Delta$ is to be accepted if and only if it results from iterated applications of the clauses of $\Psi$ to sequents having atomic arithmetical truths in their consequent, or atomic arithmetical falsities in their antecedent. This option is naturally associated with $\mathsf {I}_{\Psi }$, since it follows the idea of *grounded truth*, associated to the least Kripkean fixed point for truth. In what follows, we argue that the notion of grounded validity, as articulated by $\mathsf {I}_{\Psi }$, addresses Field’s challenge of finding a coherent reading for Beall and Murzi’s principles for naïve validity. We should stress, however, that we are not endorsing naïve validity. Our claim is simply that it can be made sense of, via grounded validity, especially if one can already make sense of the Kripkean notion of grounded truth.

#### The naïve principles for validity

We now review the case for $\mathsf {VP}$, $\mathsf {VDm}$, $\mathsf {V}$-$\mathsf {Schema}$, and $\mathsf {V}$-$\mathsf {Schema}^{+}$, construing naïve validity as grounded validity. In doing so, we also address some of Field’s more specific objections.

$\mathsf {VP}$ states that it is possible to internalise the meta-theoretical notion of naïve validity represented by $\vdash$, and express it via $\mathsf {Val}$. In the reading offered by $\mathsf {I}_{\Psi }$, $\mathsf {VP}$ says that if $\psi$ follows from $\varphi$ on the basis of arithmetical truths and falsities via the $\Psi$-clauses, then it follows on the basis of arithmetical truths and falsities via the $\Psi$-clauses that $\psi$ follows from $\varphi$ on the basis of arithmetical truths and falsities via the $\Psi$-clauses. This much is obvious, since the $\Psi$-clauses themselves include a version of $\mathsf {VP}$, that lets one express via $\mathsf {Val}$ at level $\alpha +1$ the $\vdash$-inferences accepted at level $\alpha$. This arguably answers Field’s worry that there might be no reasons to accept a ‘double occurrence’ of the notion of naïve validity on the right of $\mathsf {VP}$. Field also asks why couldn’t there be true validity claims that are not valid. While $\mathsf {I}_{\Psi }$ does not exclude this possibility, it nevertheless shows that there is a uniform construal of $\vdash$ and $\mathsf {Val}$ under which this is admissible. True *grounded*-validity claims are themselves *groundedly* valid, since grounded validity just consists in the iterative generation of all the validities that derive from our acceptance of arithmetical truths and falsities.

The justification of the $\mathsf {V}$-$\mathsf {Schema}$ follows similar lines. We have already seen how $\mathsf {I}_{\Psi }$ makes it coherent to accept its direction corresponding to $\mathsf {VP}$. As for the other direction, it follows immediately from the fixed-point property of $\mathsf {I}_{\Psi }$, i.e. from the fact that the $\Psi$-clauses are to be read as an ‘if and only if’ once we reach a fixed point. We can thus reverse the claim that closes the previous paragraph: groundedly valid validity claims are *also* just true grounded-validity claims. The extra iteration of the notion of grounded validity on the right-hand side of the $\mathsf {V}$-$\mathsf {Schema}$ does not add anything substantial to the meta-theoretical grounded validity claim on its left-hand side: the $\mathsf {V}$-$\mathsf {Schema}$ just guarantees that the two expressions (meta-theoretical and object-linguistic) of the same notion (grounded validity) are equivalent.

As for the $\mathsf {V}$-$\mathsf {Schema}^{+}$, we have seen that Field rejects it with the following example:

$$\begin{aligned} \text {snow is white}&\vdash \mathsf {Val}(`\text {grass is green'}, `\text {snow is white'}). \end{aligned}$$

This inference is invalid if $\vdash$ and $\mathsf {Val}$ express *logical* validity. However, if naïve validity is grounded validity, such an inference, and the $\mathsf {V}$-$\mathsf {Schema}^{+}$ more generally, seem perfectly acceptable. To see this, suppose we start our construction for $\mathsf {I}_{\Psi }$ not from truths and falsities of arithmetic, but from truths and falsities about the colour of snow and grass. Then, it is a truth of the selected domain that snow is white, whence we should accept ‘$\vdash$ snow is white’. Since this truth can be premised on any sentence, one also gets:

$$\begin{aligned} \text{ snow } \text{ is } \text{ white, } \text{ grass } \text{ is } \text{ green } \vdash \text{ snow } \text{ is } \text{ white } \end{aligned}$$

This is clearly acceptable, if $\vdash$ stands for ‘what follows from what, starting from truths and falsities about snow and grass, via the $\Psi$-clauses’. But then,

$$\begin{aligned} \text{ snow } \text{ is } \text{ white } \vdash \mathsf {Val}(`\text {grass is green'},`\text {snow is white'}) \end{aligned}$$

is no longer implausible: it just follows from the previous claim, internalising the $\vdash$ via the predicate $\mathsf {Val}$, which expresses the same notion of validity. The other direction of $\mathsf {V}$-$\mathsf {Schema}^{+}$ follows from the fixed point property, as explained in the previous paragraph.

Finally, the acceptance of $\mathsf {VDm}$ in $\mathsf {I}_{\Psi }$ follows from the fact that $\mathsf {I}_\Psi$ is closed under clauses which essentially express all the classical meta-inferences. In the case of $\mathsf {I}_{\Psi }$, it is hard to see why some classical meta-inference should fail. Groundedly valid inferences, expressed meta-theoretically or via Val, are determined by perfectly classical claims (about arithmetical truths and falsities), so we see no plausible reason why one should not accept all the inferences that follow from applying classical patterns of reasoning to them. $\mathsf {I}_{\Psi }$ delivers all the sequents that follow from closing the initial arithmetical sequents under all the classical meta-inferences.

#### What’s rejected: reflexivity and the full $\mathsf {VD}$

A grounded conception of validity makes it coherent to restrict $\mathsf {Ref}$ and the full $\mathsf {VD}$. $\mathsf {Ref}$ and $\mathsf {VD}$ have *ungrounded instances*, namely instances that cannot be obtained from inferences having arithmetical atomic truths in their consequents, or arithmetical atomic falsities in their antecedents. In $\mathcal {L}_{V}^{a}$, or super-languages of it, such inferences crucially feature sentences which themselves encode ungrounded inferences, via the naïve validity predicate. Inferences formed with the v-Curry sentence $\pi$ are a typical example, and indeed a grounded conception of validity rejects the instance of reflexivity that involves $\pi$, i.e. $\pi \vdash \pi$.

On a grounded conception of validity, such a conclusion need not appear so far-fetched. Recalling the equivalence between $\pi$ and , the inference $\pi \vdash \pi$ can be informally glossed as follows:

- From the fact that the inference from this very sentence to $\bot$ is naïvely valid, it follows that the inference from this very sentence to $\bot$ is naïvely valid.

But if $\mathsf {Val}$-sentences are grounded in meta-theoretical inferences, $\mathsf {Val}$-sentences ultimately derive from inferences featuring arithmetical truths or falsities. That is, in order to understand the ‘$\ldots$ is valid’ used in $\pi \vdash \pi$ from the perspective of grounded validity, one must unpack validity claims, iteratively unravelling the sentences in the scope of $\mathsf {Val}$ to ultimately determine the base-language inferences from which $\pi \vdash \pi$ derives. However, in the present case such an unravelling does not lead to inferences that do not feature the validity predicate—it leads to a circular regress. We should also stress that, much like in Kripke’s construction, cases such as $\pi \vdash \pi$ are the *only* kind of instances of $\mathsf {Ref}$ that are not in $\mathsf {I}_{\Psi }$. The case of $\mathsf {VD}$ is entirely parallel.

#### Grounded validity and Löb’s theorem

The notion of naïve validity encoded by $\mathsf {I}_{\Psi }$ would appear to avoid Field’s and Zardini’s objection from Löb’s Theorem: that $\mathsf {VD}$ and $\mathsf {VDm}$ are in conflict with Löb’s Theorem and Gödel’s Second Incompleteness Theorem. Call this the LG-objection. Running it against $\mathsf {I}_{\Psi }$ does not make much sense, since the LG-objection targets some recursively enumerable theory. However, as was mentioned at the end of Sect. 3.1, an axiomatic theory can be associated with the KV-construction, and shown to contain only sequents grounded in arithmetical axioms, thus fleshing out a weaker form of grounded validity.

Even though not every instance of  is in the so-defined axiomatic theory or in $\mathsf {I}_{\Psi }$, the following one is:4This can be thought to create a tension with Gödel’s Second Incompleteness Theorem *if* $\mathsf {Val}$ *is interpreted as a notion of provability*. However, *grounded validity* does not lend itself to such a reading. For one thing, it does not satisfy all of the Hilbert–Bernays conditions, which are constitutive of (standard) provability predicates.^21^ For another, given the defining conditions of Val in the KV-construction, Val is better understood as an implication predicate, since it has the same clauses as the classical material conditional. But the classical material conditional exceeds provability in many ways. For instance, while *modus ponens* 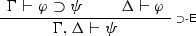 is arguably constitutive of $\supset$, the corresponding meta-inference is unacceptable for provability-in-*S*.

The notion of grounded validity provides a possible way of expressing the material conditional as an implication predicate in the object-language. Because of the v-Curry and related paradoxes, some principles that hold for the classical material conditional must be abandoned—in the case of grounded validity, reflexivity. At the same time, however, grounded validity is characterised by some principles that are constitutive of the material conditional *but not* of provability, such as VDm, which is a version of *modus ponens*. For this reason, grounded validity and provability overlap, but are not even extensionally identical.

Even if grounded validity could be interpreted as a notion of provability, the LG-objection would not have much force, since it would validate a parallel objection against non-classical theories that validate the naïve truth rules or the T-Schema. If $\mathsf {VDm}$ and (4) are to be dismissed on the grounds that they are in tension with Löb’s Theorem, it might be retorted that the naïve truth rules or the T-Schema *also* violate classical limitative results. After all, it is hard to see how (4) could be in tension with Gödel’s Second Incompleteness theorem while claiming that5(where $\lambda$ designates a Liar sentence) is not in tension with Tarski’s Theorem.

In the case of non-classical, naïve theories of truth, a standard reply is that such theories employ a non-classical logic, and hence do not violate classical limitative results. But *the same holds for grounded validity*: it might be argued that just like the conditional of (5) has to be non-classical, so too must the sequent arrow ($\vdash$) in (4). Therefore, either the LG-objection fails to apply to irreflexive, grounded validity, or structurally similar objections apply to naïve truth, thus allowing one to conclude that we should ‘have come to terms with’ the rejection of naïve truth ‘long ago’.

## Concluding remarks

Field (2017) claims that if a construction modelled after Kripke’s cannot be done that delivers Beall and Murzi’s principles,

we have a further respect in which the situation with the validity principles $\mathsf {VP}$ and $\mathsf {VD}$ seems totally different from the situation with the principles of naive truth. (Field 2017, p. 16)

We hope to have shown that such a construction can be done and that, *pace* Field, the cases of truth and naïve validity are not ‘totally different’. The naïve notion of grounded validity appears to indicate that truth and naïve validity not only give rise to similar paradoxes, but can also be understood in similar ways. Then, the resulting paradoxes can be dealt with in a similar fashion. As in the case of the paradoxes of truth, a revisionary resolution of the paradoxes of naïve validity calls for an appropriate non-classical logic, and for a coherent reading for the naïve semantic principles involved. We hope to have provided both.

## Footnotes

1 Sentences such as $\pi$ can be shown to exist in a number of ways, both in formal and natural languages. For present purposes, we simply assume their existence.

2 See Ketland (2012), Cook (2014) and Nicolai and Rossi (2017, §2).

3 See Murzi and Rossi (2017a, b).

4 For a recent discussion of the distinction between structural and substructural theories of naïve semantic notions, see Shapiro (2017). For some arguments in favour of a non-contractive approach to naïve validity, see Weber (2014).

5 A *multiset* is a collection of objects that is just like a set, except that repetitions count. Thus, for instance, $\{\varphi , \psi , \psi \}$ and $\{\varphi , \psi \}$ are identical sets but different multisets.

6 For more details on this formalism, see e.g. Troelstra and Schwichtenberg (2000, p. 41 and ff).

7 Field formulates these principles by means of a unary validity predicate, and calls (the resulting versions of) $\mathsf {NEC}$ and $\mathsf {FACT}$, respectively, $\mathsf {VALP}$ and $\mathsf {VALD}$ (Field 2017, p. 7). We stick to the binary validity predicate and employ the constant $\top$ for this reason. Moreover, we adapt Field’s principles to our framework.

8 The following derivations make also tacit use of the rules for intersubstitutivity of equivalents (e.g., in the passage labelled ‘Definition of $\sigma$’). We will always assume intersubstitutivity of equivalents without making it explicit amongst our rules for the sake of readability.

9 For more on how $\mathsf {VP}$ and $\mathsf {VDm}$ are generalisations of, respectively, $\mathsf {NEC}$ and $\mathsf {FACT}$ see Murzi and Shapiro (2015, §2.1).

10 See e.g. Kripke (1975), Field (2008), Halbach and Horsten (2006) and Horsten (2009).

11 See e.g. Asenjo (1966), Priest (1979, 2006) and Beall (2009).

12 To be precise, Field does not explicitly address $\mathsf {VDm}$. However, since he never considers restrictions of reflexivity, and $\mathsf {VD}$ is derivable from $\mathsf {VDm}$ given $\mathsf {Ref}$, we will treat $\mathsf {VD}$ and $\mathsf {VDm}$ as equivalent until Sect. 2 included (the difference between $\mathsf {VD}$ and $\mathsf {VDm}$ will only come into play in Sect. 3). Accordingly, we will interpret Field as rejecting both pairs $\mathsf {VP}$ and $\mathsf {VDm}$, and $\mathsf {VP}$ and $\mathsf {VD}$.

13 See also (Beall (2009), §2.4), Beall and Murzi (2013) and Murzi and Shapiro (2015).

14 The study of the progressions of theories resulting from the systematic addition of schematic principles (such as consistency statements, reflection principles, and others) to a starting theory was pioneered by Turing (1939). Their systematic investigation was started by Kreisel (1960, 1970) and Feferman (1962), leading to the crucial notion of *autonomous progression* (see also Feferman 1964, 1968). For an accessible presentation of progressions of theories by iterated addition of reflection principles, see Franzen (2004).

15 A well-understood example is provided by the theory of ramified analysis up to the the Feferman-Schütte ordinal $\Gamma _{0}$ (see Feferman 1964, pp. 20–21).

16 For a study of Tarski hierarchies for truth, their relation with recursive progressions of theories, and their models, see Halbach (1996, 1997); for an axiomatic presentation, see Halbach (2014, Ch. 9.1).

17 Toby Meadows (2014) also offers a Kripke-style construction for naïve validity. A proper assessment of Meadows’ construction would lead us too far afield. Here we limit ourselves to observe that (i) the construction is extremely weak from the structural standpoint, since it forces restrictions of each of the classical structural rules (reflexivity, contraction, and cut) and that (ii) it is not clear whether it addresses Field’s challenge. For a strengthening of Meadows’ theory, see Pailos and Tajer (2017).

18 Our discussion here presupposes familiarity with Kripke’s theory. For a technically detailed presentation of Kripke’s theory (strong Kleene version), see McGee (1991, Chapters 3 and 4); for a less technical presentation, see Soames (1999, Chapters 4 and 5).

19 The clause for introducing $\forall$ on the right is an $\omega$-rule. This choice was made to make the KV-construction into a genuine generalisation of Kripke’s construction. Moreover, in order to simplify the construction, we don’t include a clause for negation. A negation connective obeying the classical meta-inferences is definable from $\mathsf {Val}$, putting $\lnot \varphi$ as .

20 See Kripke (1975), p. 694 and p. 701. For an analysis of Kripkean groundedness, see Yablo (1982). For more on Kripkean grounded truth, see Leitgeb (2005), Martin (2011) and Burgess (2014).

21 See Boolos (1993). In particular, it cannot satisfy the Val-theoretic version of the second 4-like Hilbert–Bernays condition, namely .

## References

[CR1] Asenjo FG (1966). A calculus of antinomies. Notre Dame Journal of Formal Logic.

[CR2] Beall J (2009). Spandrels of truth.

[CR3] Beall J, Murzi J (2013). Two flavors of Curry’s paradox. The Journal of Philosophy.

[CR4] Boolos G (1993). The logic of provability.

[CR5] Burgess J, Tennant N (2014). Friedman and the axiomatization of Kripke’s theory of truth. Foundational adventures. Essays in honour of Harvey Friedman.

[CR6] Cook R (2014). There is no paradox of logical validity. Logica Universalis.

[CR7] Curry H (1942). The inconsistency of certain formal logics. Journal of Symbolic Logic.

[CR8] Dummett M (1959). Truth. Proceedings of the Aristotelian Society.

[CR9] Feferman S (1962). Transfinite recursive progressions of axiomatic theories. Journal of Symbolic Logic.

[CR10] Feferman S (1964). Systems of predicative analysis. Journal of Symbolic Logic.

[CR11] Feferman S (1968). Systems of predicative analysis, II. Journal of Symbolic Logic.

[CR12] Field H, Beall J (2007). Solving the paradoxes, escaping revenge. Revenge of the Liar.

[CR13] Field H (2008). Saving truth from paradox.

[CR14] Field H (2017). Disarming a paradox of validity. Notre Dame Journal of Formal Logic.

[CR15] Fischer M, Halbach V, Kriener J, Stern J (2015). Axiomatizing semantic theories of truth?. The Review of Symbolic Logic.

[CR16] Franzen T (2004). Transfinite progressions: A second look at completeness. Bulletin of Symbolic Logic.

[CR17] Halbach V (1996). Tarski-hierarchies. Erkenntnis.

[CR18] Halbach V (1997). Tarskian and Kripkean truth. Journal of Philosophical Logic.

[CR19] Halbach V (2014). Axiomatic theories of truth.

[CR20] Halbach V, Horsten L (2006). Axiomatizing Kripke’s theory of truth. Journal of Symbolic Logic.

[CR21] Horsten L (2009). Levity. Mind.

[CR22] Kaplan D, Montague R (1960). A paradox regained. Notre Dame Journal of Formal Logic.

[CR23] Ketland J (2012). Validity as a primitive. Analysis.

[CR24] Kreisel, G. (1960). Ordinal logics and the characterization of the informal concept of proof. In *Proceedings of the international congress of mathematicians (Edinburgh, 1958)* (pp. 289–299). New York: Cambrdige University Press.

[CR25] Kreisel G, Kino A, Myhill J, Vesley RE (1970). Principles of proof and ordinals implicit in given concepts. Intuitionism and proof theory.

[CR26] Kripke S (1975). Outline of a theory of truth. Journal of Philosophy.

[CR27] Leitgeb H (2005). What truth depends on. Journal of Philosophical Logic.

[CR28] Martin DA (2011). Field’s saving truth from paradox: Some things it doesn’t do. Review of Symbolic Logic.

[CR29] McGee V (1991). Truth, vagueness, and paradox.

[CR30] Meadows T (2014). Fixed-points for consequence relations. Logique et Analyse.

[CR31] Murzi J (2014). The inexpressibility of validity. Analysis.

[CR32] Murzi, J., & Rossi, L. (2017a). Generalised revenge (Unpublished manuscript).

[CR33] Murzi, J., & Rossi, L. (2017b). Reflection principles and the Liar in context (Forthcoming in Philosophers’ Imprint).

[CR34] Murzi J, Shapiro L, Achourioti T, Fujimoto K, Galinon H, Martinez-Fernandez J (2015). Validity and truth-preservation. Unifying the philosophy of truth.

[CR35] Myhill J (1960). Some remarks on the notion of proof. Journal of Philosophy.

[CR36] Nicolai C, Rossi L (2017). Principles for object-linguistic validity: From logical to irreflexive. Journal of Philosophical Logic.

[CR37] Pailos F, Tajer D (2017). Validity in a dialetheist framework. Logique et Analyse.

[CR38] Priest G (1979). The logic of paradox. Journal of Philosophical Logic.

[CR39] Priest G (2006). In contradiction.

[CR40] Ripley D (2012). Conservatively extending classical logic with transparent truth. Review of Symbolic Logic.

[CR41] Shapiro, L. (2017). The very idea of a substructural approach to paradox (Forthcoming in Synthese).

[CR42] Soames S (1999). Understanding truth.

[CR43] Troelstra AS, Schwichtenberg H (2000). Basic proof theory.

[CR44] Turing A (1939). Systems of logic based on ordinals. Proceedings of the London Mathematical Society.

[CR45] Weber Z (2014). Naïve validity. Philosophical Quarterly.

[CR46] Yablo S (1982). Grounding, dependence, and paradox. Journal of Philosophical Logic.

[CR47] Zardini E (2011). Truth without contra(di)ction. Review of Symbolic Logic.

[CR48] Zardini E (2013). Näive logical properties and structural properties. The Journal of Philosophy.

